# Post-Translationally Regulated Protein Arginine-to-Proline Conversion in Alzheimer’s Brains

**DOI:** 10.3390/life12070967

**Published:** 2022-06-28

**Authors:** Yuichiro Justin Suzuki

**Affiliations:** Department of Pharmacology and Physiology, Georgetown University Medical Center, Washington, DC 20007, USA; ys82@georgetown.edu

**Keywords:** amino acid conversion, arginine-to-proline conversion, oxidant, post-translational regulation, protein, reactive oxygen species, redox

## Abstract

The current belief is that amino acid sequences in protein structures are defined by DNA sequences. I challenge this concept by hypothesizing that an arginine (Arg) residue in the protein structure can post-translationally be converted to a proline (Pro) residue through a redox mechanism. Reactive oxygen species promote the formation of protein carbonylation, particularly on Arg and Pro residues, which both produce glutamyl semialdehyde. Our previous studies suggested that the Pro-to-glutamyl semialdehyde reaction could be reversible in the biological system, thereby opening up a pathway for the conversion of Arg to glutamyl semialdehyde by oxidation, and subsequently, to Pro by reduction in the protein structure. Our mass spectrometry and immunoblotting experiments provided evidence of the occurrence of the Arg-to-Pro conversion at position 108 (R108P) of the peroxiredoxin 6 (Prx6) protein in biological tissues and cells. In the human brain, Prx6 (R108P) occurs, and some Alzheimer’s brains exhibit increased Prx6 (R108P) levels, while others show decreased levels, indicating the complexity of redox processes in the disease state. I propose that Prx6 (R108P), as well as other post-translationally regulated protein Arg-to-Pro conversions, occur in the human body and play physiological and pathological roles.

## 1. Introduction

The current belief is that the amino acid sequences in protein structures are defined by DNA sequences [1]. Alternations in amino acid sequences occur in response to DNA mutation and could have significant impacts in pathophysiology [2]. As a molecular tool, site-directed mutagenesis can change any amino acid residues by mutating DNA sequences [3]. In this article, I pose a hypothesis that such changes in amino acid sequences occur post-translationally in the biological system through oxidation and reduction reactions. Such processes should have serious implications for the pathogenesis of various diseases, including Alzheimer’s disease.

Alzheimer’s disease is a chronic neurodegenerative disorder and the main cause of dementia. It affects a large number of older individuals, with an estimated prevalence of 10–30% in the population >65 years of age [4]. In the United States, an estimated 5.8 million people have Alzheimer’s disease and it is the fifth leading cause of death among older adults [5]. However, little is known about the cause of Alzheimer’s disease and no curative treatments are available. Thus, further understanding the mechanisms of the development and progression of Alzheimer’s disease is warranted. Oxidative stress has been implicated in the pathogenesis of Alzheimer’s disease as well as in aging [6,7,8].

**Figure 1:**
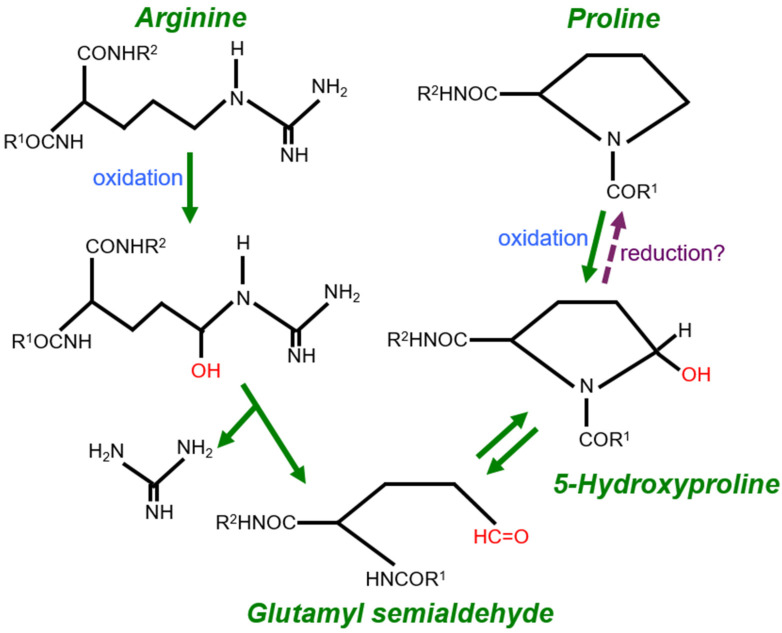
Oxidation of Arg and Pro residues and possible conversion of Arg to Pro in the protein structure. Oxidation of both Arg and Pro residues of proteins forms glutamyl semialdehyde with a carbonyl group in a process called protein carbonylation. From studies of purified proteins, it is thought that Arg-to-glutamyl semialdehyde as well as Pro-to-5-hydroxyproline reactions are irreversible, while the 5-hydroxyproline-to-glutamyl semialdehyde reaction is reversible [12]. However, our studies provided evidence that cells possess a catalytic ability to reduce 5-hydroxyproline to Pro [13,14], suggesting the possibility that Arg becomes Pro in the protein structure post-translationally in the biological system.

Oxidative stress occurs in response to the production of reactive oxygen species (ROS), which can damage DNA, protein, lipids, and small molecules [9]. Protein carbonylation refers to two types of iron-catalyzed protein oxidation promoted by ROS. Primary protein carbonylation involves the direct oxidation of amino acid residues, while secondary protein carbonylation occurs in response to the incorporation of lipid-derived aldehydes [10]. Arginine (Arg), proline (Pro), lysine, and threonine have been reported to be the major amino acid residues subjected to primary protein oxidation [11,12]. The oxidation of both Arg and Pro residues in the proteins produces glutamyl semialdehyde that contains a carbonyl group, as depicted in Figure 1.

Stadtman and co-workers, through their studies on purified proteins, proposed that both Arg-to-glutamyl semialdehyde and Pro-to-glutamyl semialdehyde reactions are irreversible as indicated with green arrows in Figure 1 [12]. However, cell research in my laboratory resulted in the discovery of the protein decarbonylation process [13], and the identification of a biological catalyst that can promote protein decarbonylation [14] led me to an idea that, in the biological system, the Pro-to-glutamyl semialdehyde reaction can be reversed through catalyzed reduction reactions (the purple arrow in Figure 1).

Thus, I propose that an Arg residue can become a Pro residue within the protein structure (namely the Arg-to-Pro conversion) through the oxidant-mediated production of glutamyl semialdehyde. This results in outcomes accomplished by protein engineering/site-directed mutagenesis, but in this case, through a naturally-occurring post-translationally regulated mechanism.

## 2. The Hypothesis

My hypothesis is that the Arg-to-Pro conversion within the protein structure can occur in the biological system that is regulated post-translationally through mechanisms involving redox reactions. This hypothesis is based on the knowledge that both Arg and Pro residues are oxidized and become glutamyl semialdehyde and our experimental observations that the Arg108-to-Pro substituted peroxiredoxin 6 (Prx6) protein occurs in human cells and tissues, including Alzheimer’s brains. This hypothesis is important because it suggests that the protein sequences are not strictly defined by DNA sequences, opening up the possibility that proteins with altered sequences may play physiological roles.

## 3. Evaluation of the Hypothesis

Our laboratory previously discovered that ligand/receptor-mediated cell signaling promotes protein carbonylation in cultured cells [13]. Interestingly, the observed carbonyl formation was found to be transient, with the peak occurring about 10 min after the ligand treatment of cells. This time course is similar to ligand/receptor-mediated protein phosphorylation and subsequent dephosphorylation. Thus, we termed the phase of decreased carbonyl content “protein decarbonylation” [13]. The protein phosphorylation system is defined by the balance between protein kinases and phosphatases, and protein dephosphorylation is promoted by protein phosphatases. However, it was not clear how protein decarbonylation would be regulated. We hypothesized that protein decarbonylation may be promoted by electron reduction and, interestingly, found that thiol reductants can decrease the levels of protein carbonylation [13]. Moreover, this thiol reductant effect on protein carbonylation does not occur in purified proteins, but only in the presence of biological components, such as in tissue homogenates and cell lysates [14]. We further identified that glutaredoxin 1 is one protein that regulates protein decarbonylation [14]. These findings suggested that biological catalysts, such as glutaredoxin 1, may reduce glutamyl semialdehyde to Pro in the protein structure, revealing the possibility of the occurrence of the protein Arg-to-Pro conversion.

Since the role of glutaredoxin 1 was found while we were studying the regulation of carbonylation and decarbonylation in the Prx6 protein, we performed mass spectrometry experiments to identify the possible occurrence of the Arg-to-Pro conversion in growth-factor-treated cells. Results revealed that Arg 108 could be converted to a Pro residue in a small percentage of overall Prx6 molecules. We generated an antibody that specifically detects Prx6 with Arg 108 substituted with Pro and tested if this approach allows for the detection of Arg108-to-Pro-converted Prx6 protein molecules. This antibody was generated by Innovagen (Lund, Sweden) by immunizing rabbits with the (NH2-) CFPIIDDRNPELAILL (-CONH2) peptide. The mutation-specific antibody interacted with the Prx6 (100–114) R108P peptide 5.2-times more strongly than with the Prx6 (100–114) peptide.

**Figure 2:**
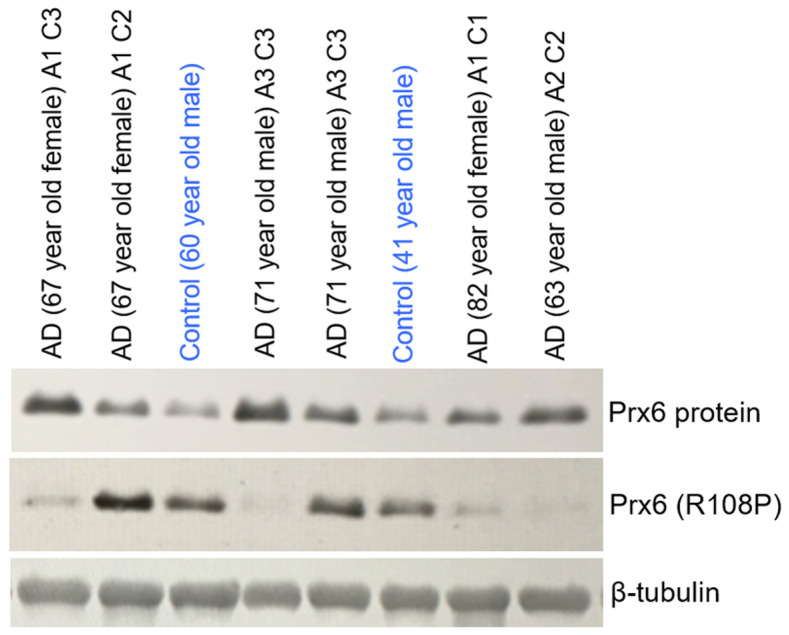
The expression of wild-type Prx6 and Prx6 with Arg108 substituted with Pro (R108P) in brains of Alzheimer’s disease (AD) patients and controls. Brain (hippocampus) tissues of Alzheimer’s disease patients and control subjects without Alzheimer’s disease or other neurological diseases [15] were homogenized and subjected to Western blotting using a Prx6 protein antibody (MilliporeSigma, Burlington, MA, USA), a custom-made antibody that specifically detects Prx6 (R108P) (Innovagen), and a β–tubulin antibody (Sino Biological, Beijing, China) as a loading control. Age, gender, Aβ plaque scores (A1–A3), and neuritic plaque scores (C1–C3) [15] of these patients and control subjects are indicated.

Figure 2 shows our immunoblotting results using this Prx6 (R108P) antibody on human brain (hippocampus) tissue samples of patients with Alzheimer’s disease, as well as control subjects without known neurological disorders. Results shown in lanes 3 and 6 reveal that the brain tissues from the control subjects exhibit positive signals for the Arg108-to-Pro-converted Prx6 protein. We previously reported that Alzheimer’s brains consistently have increased levels of Prx6 protein compared with controls [15]. This may reflect an adaptive response by increasing the level of the antioxidant defense mechanism in response to increased oxidative stress in Alzheimer’s brains [15], as Prx6 is an antioxidant enzyme that eliminates peroxides [16,17]. Figure 2 also shows that Alzheimer’s disease brains consistently have higher Prx6 protein levels than controls. By contrast, the levels of Prx6 with Arg108-to-Pro substitutions are higher in some Alzheimer’s disease patient brains and lower in others. Alzheimer’s disease brains in lanes 1, 4, 7, and 8 have lower Prx6 (R108P) levels, despite the Prx6 protein levels being higher than those of the controls.

These results provided evidence that support the occurrence of the Arg-to-Pro conversion in the human brain. It is interesting, however, that the level of amino-acid-converted protein does not directly follow the degree of oxidative stress. This is reasonable, if we consider that high oxidative stress hinders the reductive process that drives the formation of Pro from glutamyl semialdehyde.

The limitation of these immunological studies includes the possibility that the detected Arg108-to-Pro conversion may be a result of DNA mutation or RNA editing rather than the post-translationally regulated process. Thus, further studies are needed to prove the occurrence of the post-translationally regulated Arg-to-Pro conversion.

## 4. Consequences of the Hypothesis

I describe here the Arg-to-Pro conversion within the protein structure that occurs post-translationally through a redox mechanism. The occurrence of post-translationally regulated protein amino acid conversions, including the Arg-to-Pro conversion, should revolutionize protein biochemistry. Because of advances in molecular biology, the protein sequence information generated since the 1980s has all been deduced from DNA sequences, and we, thus, assume that DNA sequences strictly govern protein sequences. Since we have stopped performing amino acid sequencing to deduce the primary structures of proteins, whether altered sequences from DNA sequences exist in proteins in the biological system is unknown, as is whether such altered sequences regulate biological functions. Conducting further studies of whether oxidant-mediated protein amino acid conversions within the protein structure occur in the biological systems and testing whether proteins whose amino acid sequences deviate from the DNA sequences play roles in pathophysiology should dramatically advance biology and help develop improved therapeutic strategies against many diseases.

## 5. Conclusions

As the biological system may use redox reactions to convert one amino acid to another, site-directed mutagenesis-like events could occur post-translationally. The oxidation of Arg residues produces glutamyl semialdehyde and the biological system may possess catalysts to reduce glutamyl semialdehyde to Pro. Then, oxidation-to-reduction reactions could convert Arg residues to Pro residues in the protein structure. This hypothesis is based on our previous discoveries of the protein decarbonylation process [13] and existence of biological catalysts that promote protein decarbonylation [14], as well as my preliminary mass spectrometry and immunoblotting results showing the occurrence of Arg108 substitution with Pro on the Prx6 protein molecule in biological tissue and cell samples, including human brains from Alzheimer’s disease patients.

## Data Availability

The data presented in this study are available on request from the corresponding author.
